# Effect of COPD on symptoms, quality of life and prognosis in patients with advanced non-small cell lung cancer

**DOI:** 10.1186/s12885-018-4976-3

**Published:** 2018-10-29

**Authors:** Young-Soo Yi, Woo Ho Ban, Kyeong-Yae Sohng

**Affiliations:** 1 Doctoral candidate, The Catholic University of Korea, Seoul, South Korea; 2 Division of pulmonary, Critical Care and sleep Medicine, Department of Internal Medicine, College of Medicine, The Catholic University of Korea, Seoul, South Korea; 3 Department of Nursing, College of Nursing, The Catholic University of Korea, Seoul, South Korea

**Keywords:** Pulmonary disease, Chronic obstructive, Quality of life, Lung neoplasms

## Abstract

**Background:**

Many studies have reported the prevalence of chronic obstructive pulmonary disease (COPD) and its effects and prognosis in patients with lung cancer, but few have considered quality of life and survival of patients with lung cancer according to severity of airway obstruction. This study investigated the presence of COPD and the severity of airway obstruction in patients with non-small cell lung cancer (NSCLC), and analyzed how these factors affected symptoms, quality of life, and prognosis.

**Methods:**

We retrospectively reviewed the prospective lung cancer database of the Catholic Medical Centers at the Catholic University of Korea from 2014 to 2017. We enrolled patients with advanced NSCLC and evaluated quality of life using the European Organization for Research and Treatment of Cancer Quality of Life Questionnaire-C30. We also estimated pulmonary function and analyzed survival data.

**Results:**

Of the 337 patients with advanced NSCLC, 170 (50.5%) had COPD and 167 (49.5%) did not. Significant differences were observed in symptoms between the two groups. The COPD group complained of more symptoms, such as cough, sputum, and dyspnea, than those in the non-COPD group. The distribution according to the severity of obstruction in the COPD group was as follows: Grade 1 (FEV_1_ ≥ 80%) 35 patients (20.6%), Grade 2 (50% ≤ FEV_1_ < 80%) 103 patients (60.6%), Grade 3 (30% ≤ FEV_1_ < 50%) 24 patients (14.1%), and Grade 4 (FEV_1_ < 30%) 8 patients (4.7%). The presence of COPD did not affect overall quality of life in patients with NSCLC, but as the airway obstruction increased, physical function decreased, and fatigue and dyspnea were more frequent. The overall median survival of the COPD group was shorter than that of the non-COPD group (median survival, 224 vs. 339 days, *p* = 0.035).

**Conclusions:**

In this study, a high prevalence of COPD was found among patients with advanced NSCLC, and COPD patients complained about various symptoms and had diminished quality of life in several sectors. Therefore, it is necessary to actively evaluate quality of life, lung function, and symptoms in patients with lung cancer and reflect them in the treatment and management plans of these patients.

## Background

Lung cancer is a disease with high incidence and mortality rates worldwide. The American Cancer Society estimated that in the United States, 162,510 people died of lung cancer in 2016 [1]. In Europe, the socioeconomic costs due to lung cancer amounted to 18.8 billion Euros in 2009, which is about 15% of the total cost of cancer in the region [2]. Thus, lung cancer is considered to be one of the most economically burdensome diseases at the national level. At the individual level, lung cancer often causes not only general symptoms, such as pain and fatigue but also various respiratory symptoms, including cough, sputum, and dyspnea. Additionally, as lung cancer patients primarily have extensive-stage disease at the time of diagnosis, they tend to have significant mental difficulties, such as fear of death, depression, and anxiety [3].

The main cause of lung cancer is smoking [4], and chronic obstructive pulmonary disease (COPD) is typically caused by smoking. Therefore, COPD and lung cancer have a close relationship in terms of prevalence and symptoms reported by patients. COPD is an independent risk factor for lung cancer, and smokers with an obstruction have more than five times the incidence of lung cancer than those with normal lung function [5]. Cough, sputum, and dyspnea are the most common respiratory symptoms experienced by patients with COPD, and these symptoms can reduce quality of life. Similarly, various symptoms, including dyspnea and quality of life, affect the prognosis of patients with lung cancer. In particular, respiratory symptoms and changes in pulmonary function, which serve as diagnostic criteria for COPD, have a very negative impact on the prognosis of patients with lung cancer [6–8]. Many studies have reported the prevalence of COPD and its effects and prognosis in patients with lung cancer, but few have considered quality of life and survival of patients with lung cancer according to severity of airway obstruction. Therefore, this study examined the presence of COPD in patients with advanced non-small cell lung cancer (NSCLC), investigated differences in quality of life and symptoms according to severity of airway obstruction, and analyzed how these factors affect prognosis.

## Methods

### Study population and data collection

A retrospective study of patients enrolled in the Catholic Medical Centers (CMC) lung cancer registry run by Catholic University (CMC lung cancer registry) was conducted from October 2014. The CMC lung cancer registry was developed to investigate the relationship between initial clinical parameters and outcomes through the serial observation in patients with lung cancer. The initial symptoms and quality of life through questionnaire survey, clinical characteristics, smoking history, comorbidities, pulmonary function tests, histological and cytological diagnosis, staging and treatment outcomes were collected prospectively and systematically from the time of diagnosis to death. Among the patients who were histologically and cytologically diagnosed with NSCLC, 337 with stage-3 and stage-4 NSCLC were selected for the study based on the Seventh Edition of the American Thoracic Society Tumor Node Metastasis classification [9–12]. Quality of life was evaluated using the European Organization for Research and Treatment of Cancer Quality of Life Questionnaire (EORTC QLQ)-C30 (Korean version) [13–15]. The EORTC QLQ-C30 was found to be a reliable and valid measure of reported health-related quality of life [16]. The validity and reliability of the EORTC QLQ-C30 (Korean version) has been reported in previous study (Cronbach’s α > 0.7) [17]. The EORTC QLQ-C30 is composed of multi-item and single scales: five functional scales (physical, role, emotional, cognitive and social), three symptom scales (fatigue, nausea & vomiting and pain), six single items (dyspnea, insomnia, appetite loss, constipation, diarrhea and difficulties) and a global health status/QOL scale [15]. All scales and items are linearly transformed to 0–100 scale [18]. For the 5 functional scales and the global quality of life scale, a higher score represents a better level of functioning. For the symptom scales and items, a high score corresponds to a higher level of symptom [18]. The diagnosis of COPD was made when the ratio of forced expiratory volume in 1 s (FEV_1_) to forced vital capacity (FVC) was < 70% based on the 2014 GOLD (Global Initiative for Chronic Obstructive Lung Disease) guidelines. The distribution of patients with COPD according to severity of the obstruction was classified into Grade 1 (FEV_1_ ≥ 80%), Grade 2 (50% ≤ FEV_1_ < 80%), Grade 3 (30% ≤ FEV_1_ < 50%), and Grade 4 (FEV_1_ < 30%) [19, 20]. Data regarding patients’ age, sex, smoking history, histological type and the Eastern Cooperative Oncology Group Performance Status Scale (ECOG PS) were also obtained from the registry. The ECOG PS is widely used to assess the functioning of cancer patients specifically for the purposes of oncological decision making, as well as prediction of patients’ prognosis. It provides a five-point scale which incorporates elements such as ambulatory status and need for care [21]. A survival analysis was conducted based on the last date of tracking observations (June 30, 2017). This study was approved by the Clinical Research Ethics Committee of the Catholic Medical Center (XC140IMI0070).

### Statistical analysis

Descriptive statistics were used to summarize patients’ demographic characteristics, symptoms and QLQ score. Data were analyzed for statistical significance using the Pearson’s chi-square test or unpaired t-test for patient’s characteristics and QLQ scores between the COPD group and non COPD group, the Kruskal-Wallis test for QLQ scores between FEV_1_ groups. Additionally, Cochran –Mantel -Haenszel statistics were used to test for associations, and generalized linear models were used to test for a linear trend. Survival curves according to COPD and non- COPD were drawn using the Kaplan- Meier Method, and compared by the log-rank test. Hazard ratios (HRs) and 95% confidence intervals (CIs) were calculated with the Cox proportional hazards model to identify independent prognostic factors. The factors significantly associated with patient survival in univariate analysis (*p* < 0.05) were included in multivariate analysis. All analyses were performed with the use of SAS (version 9.4; SAS Institute, Cary, NC, USA) or R (version 3.4.1; R Computing, Vienna, Austria). A p -value < 0.05 was considered significant.

## Results

### Clinical characteristics

A total of 337 patients were enrolled in this study and were classified into two groups according to the presence of COPD. The general and clinical characteristics of the subjects are as follows (Table 1). Among the subjects, the number of patients with COPD (FEV_1/_FVC < 70) was 170 (50.5%), and the number without (FEV_1/_FVC ≥ 70) was 167 (49.5%). The average age of the COPD group was 70.4 years, and that of the non-COPD group was 63.2; the distribution of patients aged >65 years was significantly higher in the COPD group than that in the non-COPD group. A total of 154 males (90.6%) were in the COPD group, and 108 (64.7%) were in the non-COPD group, indicating significantly larger number compared to that of females. The distribution of smokers was significantly higher in the COPD group [152 (89.4%)], and [104 (62.3%)] than that in the non-COPD group. The histology results were significant: the COPD group had a higher proportion of squamous-cell carcinoma, whereas the proportion of adenocarcinoma was higher in the non-COPD group. In terms of comorbid conditions, the number of patients with one or more comorbidities was 103 (60.6%) in the COPD group, and 75 (44.9%) in the non-COPD group. In particular, the COPD group had higher proportions of tuberculosis [COPD vs. non-COPD; 38 (22.4%) vs. 16 (9.6%)], and hypertension [COPD vs. non-COPD; 48 (28.2%) vs. 30 (18.0%)]. The proportions of old age, smoking history, comorbidities, and squamous cell type were significantly higher in the COPD group than in the non-COPD group. The distribution of patients with COPD according to severity of the airway obstruction was Grade 1 (FEV_1_ ≥ 80%) 35 patients (20.6%); Grade 2 (50% ≤ FEV_1_ < 80%) 103 patients (60.6%); Grade 3 (30% ≤ FEV_1_ < 50%) 24 patients (14.1%); and Grade 4 (FEV_1_ < 30%) 8 patients (4.7%).

**Table 1:** Patient characteristics

Variables	Total (*n* = 337)	COPD (*n* = 170)	Non-COPD (*n* = 167)	*p* value
Age(years)				
Mean(±SD)	66.8(±10.9)	70.4(±8.9)	63.2(±11.6)	5.550E-10
≥ 65	195(57.9)	120(70.6)	75(44.9)	1.814E-06
< 65	142(42.1)	50(29.4)	92(55.1)	
Gender				
Male	262(77.7)	154(90.6)	108(64.7)	1.072E-08
Female	75(22.3)	16(9.4)	59(35.3)	
History of smoking				
Never smoker	81(24.0)	18(10.6)	63(37.7)	5.580E-09
Former or current smoker	256 (76.0)	152(89.4)	104(62.3)	
Cancer stage				
III	123(36.5)	70(41.2)	53(31.7)	0.090
IV	214(63.5)	100(58.8)	114(68.3)	
Histology				
Adenocarcinoma	179(53.1)	60(35.3)	119(71.3)	1.872E-09
Squamous-cell carcinoma	132(39.2)	94(55.3)	38(22.8)	
Large cell carcinoma	1(0.3)	1(0.6)	0(0.0)	
Adenosquamous carcinoma	2(0.6)	2(1.2)	0(0.0)	
Other	23(6.8)	13(7.7)	10(6.0)	
Comorbid conditions				
No	159(47.2)	67(39.4)	92(55.1)	0.005
Any comorbid condition	178(52.8)	103(60.6)	75(44.9)	
Comorbidities				
Tuberculosis (Active + old)	54(16.0)	38(22.4)	16(9.6)	0.002
Pneumoconiosis	5(1.5)	5(2.9)	0(0.0)	0.061
Heart disease	19(5.6)	11(6.5)	8(4.8)	0.638
Diabetes mellitus	60(17.8)	33 (19.4)	27 (16.2)	0.478
Hypertension	78(23.2)	48(28.2)	30(18.0)	0.028
Others	76(22.6)	45(26.5)	31(18.6)	0.049
FEV_1_ level				
GOLD grade1	129(38.3)	35(20.6)	94(56.3)	1.200E-11
GOLD grade2	171(50.7)	103(60.6)	68(40.7)	
GOLD grade3	28(8.3)	24(14.1)	4(2.4)	
GOLD grade4	9(2.7)	8(4.7)	1(0.6)	

Data are mean (±SD) and n (%). *COPD* chronic obstructive pulmonary disease, *FEV*_*1*_ forced expiratory volume in 1 s, *GOLD* Global Initiative for Chronic Obstructive Lung Disease

For continuous variables, Student’s t test was used; for univariate analysis, the chi-square test was used

### Symptoms of patients between the groups with and without COPD

Table 2 lists the differences in symptoms between COPD and non-COPD patients. Cough was observed in 117 patients with COPD (68.8%) and 95 patients with non-COPD (56.9%); sputum in 99 patients with COPD (58.2%) and 74 patients with non-COPD (44.3%); dyspnea in 60 patients with COPD (35.3%) and 33 patients with non-COPD (19.8%). As shown in Table 2, cough, sputum, and dyspnea were more common in patients with COPD than those without. Additionally, the number of symptoms was significantly greater in patients with COPD than in those without at the time of the lung cancer diagnosis(*p* < .0001).

**Table 2:** Symptoms of patients with and without chronic obstructive pulmonary disease (COPD)

Variables	Total (*n* = 337)	COPD (*n* = 170)	Non-COPD (*n* = 167)	*p* value
Respiratory				
Cough	212 (62.9)	117 (68.8)	95 (56.9)	0.025
Sputum	173 (51.3)	99 (58.2)	74 (44.3)	0.012
Chest pain	126 (37.4)	70 (41.2)	56 (33.5)	0.177
Hoarseness	67 (19.9)	39 (22.9)	28 (16.8)	0.173
Wheezing or stridor	88 (26.1)	49 (28.8)	39 (23.4)	0.267
Dyspnea	93 (27.6)	60 (35.3)	33 (19.8)	0.002
Hemoptysis	24 (7.1)	17 (10.0)	7 (4.2)	0.055
General				
Fatigue	144 (42.7)	74 (43.5)	70 (41.9)	0.826
Weight loss	121 (35.9)	64 (37.7)	57 (34.1)	0.570
Pain	80 (23.7)	38 (22.4)	42 (25.2)	0.609
General weakness	112 (33.2)	60 (35.3)	52 (31.1)	0.421
Poor oral intake	113 (33.5)	63 (37.1)	50 (29.9)	0.204
Fever	18 (5.3)	8 (4.7)	10 (6.0)	0.636
Number of symptoms				
Mean(±SD)	0.8(±0.7)	1.0(±0.7)	0.5(±0.6)	1.332E-13
0	55 (16.3)	17 (10.0)	38 (22.8)	0.004
1	60 (17.8)	29 (17.1)	31 (18.56)	
≥ 2	222 (65.9)	124 (72.9)	98 (58.7)	

Data are mean (±SD) and n (%). COPD, chronic obstructive pulmonary disease

For continuous variables, Student’s t test was used; for univariate analysis, the chi-square test was used

### EORTC-QLQ scores between the groups with and without COPD

The EORTC QLQ-C30 scale was analyzed according to the presence of COPD in patients with advanced NSCLC (Table 3). Overall quality of life tended to be lower in patients with COPD than in patients without (*p* > 0.05). However, cognitive and social function scales were significantly lower in the COPD group, and the severity of dyspnea and appetite loss was significantly higher on the symptom scale.

**Table 3:** European Organization for Research and Treatment of Cancer Quality of Life Questionnaire (EORTC QLQ)-C30 (Korean version) scores between patients with and without chronic obstructive pulmonary disease (COPD)

Variables	COPD (*n* = 170)	Non-COPD (*n* = 167)	*p* value
Global health status/ QoL	48.0 ± 23.7	51.1 ± 23.2	0.225
Functional scales			
Physical functioning	74.8 ± 23.2	78.8 ± 21.8	0.099
Role functioning	74.7 ± 28.3	80.4 ± 25.8	0.053
Emotional functioning	76.1 ± 25.0	78.7 ± 19.5	0.295
Cognitive functioning	80.3 ± 24.0	85.1 ± 17.6	0.036
Social functioning	68.6 ± 27.1	75.4 ± 23.3	0.014
Symptom scales/items			
Fatigue	34.1 ± 24.3	30.0 ± 24.4	0.128
Nausea and vomiting	8.1 ± 17.1	8.1 ± 16.1	0.977
Pain	21.2 ± 27.9	21.5 ± 25.5	0.923
Dyspnea	33.3 ± 32.2	23.2 ± 30.3	0.003
Insomnia	28.4 ± 30.5	23.2 ± 29.7	0.108
Appetite loss	30.8 ± 34.0	24.0 ± 28.8	0.048
Constipation	17.5 ± 28.6	20.0 ± 27.1	0.410
Diarrhea	7.5 ± 19.1	8.2 ± 17.0	0.710
Financial difficulties	32.5 ± 31.0	29.5 ± 30.5	0.370

Data are mean ± SD. *COPD* chronic obstructive pulmonary disease, *QoL* quality of life;

Student’s t test was used

### EORTC-QLQ scores of patients with COPD according to airway obstruction

Table 4 lists the EORTC QLQ-C30 results according to severity of airway obstruction in the COPD group among patients with advanced NSCLC. The overall quality of life in patients with COPD was significantly lower as the severity of obstruction increased. In particular, a significant difference in the decline of physical functioning was observed between the groups. The severity of fatigue and dyspnea increased significantly with severity of airway obstruction.

**Table 4:** European Organization for Research and Treatment of Cancer Quality of Life Questionnaire (EORTC QLQ)-C30 scores of patients with chronic obstructive pulmonary disease (COPD) according to airway obstruction

Variables	GOLD grade 1 (*n* = 35)	GOLD grade 2 (*n* = 103)	GOLD grade 3 (*n* = 24)	GOLD grade 4 (*n* = 8)	*p* value
Global health status/QoL	50.5 ± 26.3	50.2 ± 23.0	41.0 ± 19.0	30.2 ± 26.7	0.049
Functional scales					
Physical functioning	83.4 ± 17.1	75.4 ± 22.7	66.7 ± 24.4	53.3 ± 30.4	0.004
Role functioning	79.5 ± 31.3	75.9 ± 24.2	69.4 ± 33.9	54.2 ± 39.6	0.132
Emotional functioning	75.0 ± 26.2	76.4 ± 25.0	78.5 ± 21.8	70.8 ± 32.1	0.970
Cognitive functioning	87.1 ± 21.4	79.0 ± 25.1	77.8 ± 22.3	75.0 ± 21.8	0.123
Social functioning	72.4 ± 28.9	68.1 ± 26.4	68.1 ± 26.4	60.4 ± 33.3	0.646
Symptom scales/items					
Fatigue	25.1 ± 24.6	34.5 ± 22.8	40.7 ± 24.7	47.2 ± 31.3	0.011
Nausea and vomiting	9.5 ± 23.3	9.2 ± 15.8	1.4 ± 4.7	8.3 ± 23.6	0.059
Pain	18.6 ± 30.2	22.0 ± 26.8	20.1 ± 26.5	25.0 ± 38.8	0.622
Dyspnea	20.0 ± 24.5	33.7 ± 31.8	44.4 ± 36.3	54.2 ± 35.4	0.010
Insomnia	24.8 ± 31.7	27.2 ± 29.4	36.1 ± 31.0	37.5 ± 37.5	0.352
Appetite loss	27.6 ± 36.6	30.4 ± 32.0	31.9 ± 36.1	45.8 ± 43.4	0.621
Constipation	14.3 ± 24.6	16.2 ± 26.8	19.4 ± 31.0	41.7 ± 49.6	0.435
Diarrhea	7.6 ± 18.2	7.8 ± 20.5	6.9 ± 17.0	4.2 ± 11.8	0.988
Financial difficulties	25.7 ± 31.4	32.7 ± 29.9	40.3 ± 34.0	37.5 ± 33.0	0.320

Data are mean (±SD). *GOLD* Global Initiative for Chronic Obstructive Lung Disease, *QoL* quality of life

ANOVA test was used

### Effects of COPD on survival in NSCLC

**Fig. 1:**
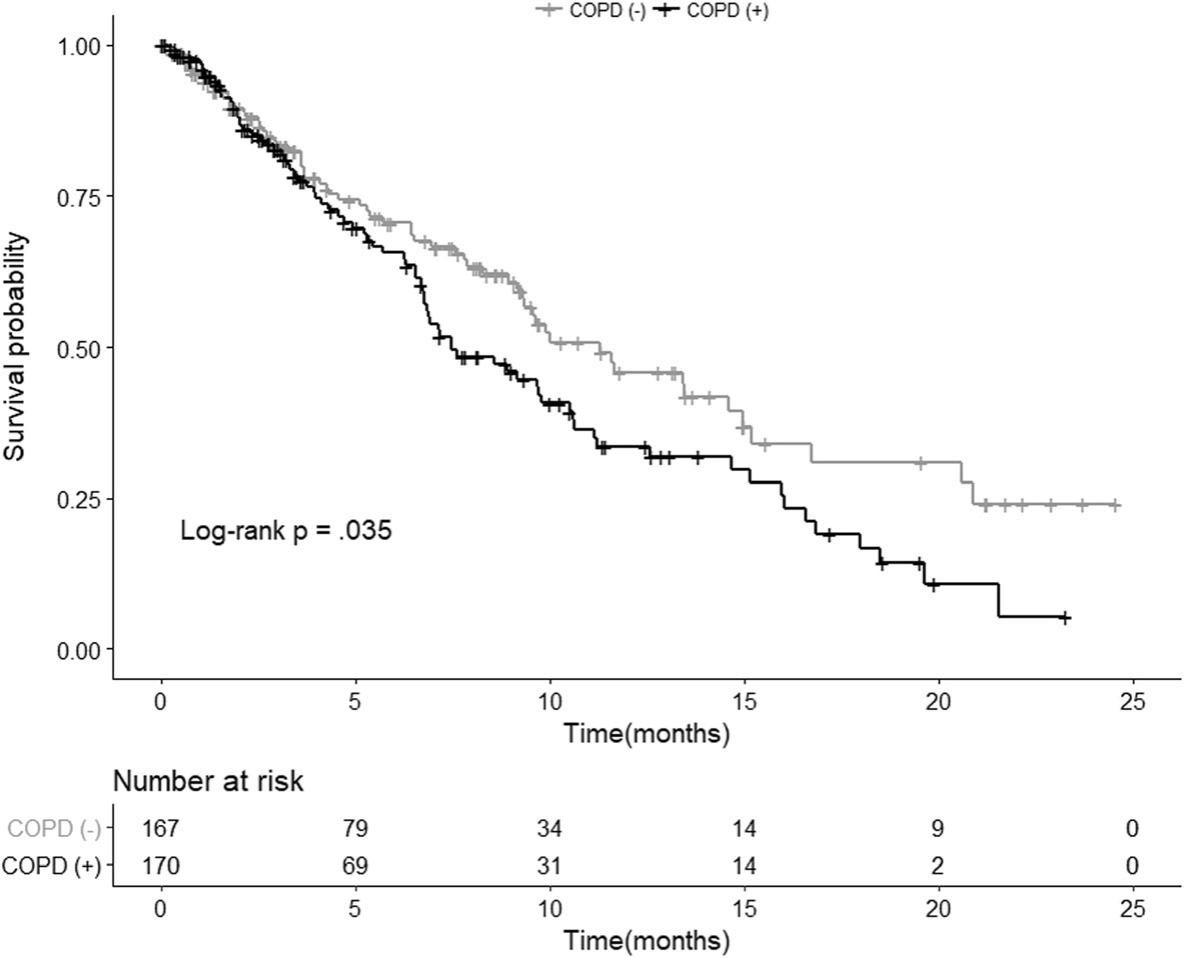
Kaplan-Meier survival curve of patients according to chronic obstructive pulmonary disease (COPD)

The results of the analysis of whether the presence of COPD affected survival of patients with advanced NSCLC are as follows (Fig. 1). The overall median survival time for all patients was 111 days. The overall median survival of the COPD group was shorter than that of the non-COPD group (median survival, 224 vs. 339 days, *p* = 0.035). A univariate analysis indicated that the presence of COPD and stage, sex, and FEV_1_ level had significant effects on patient survival. However, the multivariate analysis revealed that advanced stage (HR, 1.87; 95% CI: 1.26–2.77) and male sex (HR, 2.32; 95% CI: 1.45–3.72) were significant poor prognostic factors affecting survival in patients with NSCLC (Table 5).

**Table 5:** Survival analysis of patients with chronic obstructive pulmonary disease (COPD) according to airway obstruction

Variables	Univariate analysis		Multivariate analysis	
	HR (95% CI)	*p*-value	HR (95% CI)	*p*-value
COPD				
(−)	1		1	
(+)	1.42 (1.02–1.98)	0.036	0.94 (0.64–1.38)	0.763
Stage				
Stage 3	1		1	
Stage 4	1.68 (1.14–2.48)	0.009	2.03 (1.36–3.01)	0.001
Age				
< 65	1		1	
≥ 65	1.76 (1.22–2.53)	0.003	1.87 (1.28–2.73)	0.001
Sex				
Female	1		1	
Male	2.00 (1.28–3.12)	0.002	2.26 (1.41–3.62)	0.001
BMI				
< 23	1			
≥ 23	0.78 (0.56–1.09)	0.143		
Comorbidity				
No	1			
Yes	1.39 (0.99–1.93)	0.054		
History of smoking				
Never smoker	1			
Former or current smoker	1.51 (0.99–2.29)	0.056		
Symptom				
No	1			
Yes	1.55 (0.97–2.49)	0.070		
FEV_1_				
≥ 60	1		1	
< 60	1.46 (1.02–2.09)	0.041	1.42 (0.96–2.11)	0.084

*COPD* chronic obstructive pulmonary disease, *BMI* body mass index, *FEV*_*1*_ forced expiratory volume in 1 s, *HR* hazard ratio, *CI* confidence interval

The Cox proportional hazards modeling technique was used

## Discussion

Smoking is the major cause of lung cancer and is also known to cause various other diseases, including diabetes and cerebral cardiovascular diseases. In particular, smoking is closely related to COPD. This study investigated quality of life and symptoms of COPD and analyzed how COPD affected prognosis in patients with advanced NSCLC. The results confirmed that patients with advanced NSCLC and COPD had more symptoms and reduced quality of life in several aspects, but these did not affect the survival rate in the patients. In this study, the prevalence of COPD was 50.5% among all patients with advanced NSCLC. Several studies have focused on the frequency of COPD in lung cancer: Ytterstad et al. reported that 39% of patients with lung cancer have COPD [22]. In the present study, the prevalence rate of COPD in patients with NSCLC was relatively higher than that previous study, possibly because it excluded patients with early lung cancer who have a shorter smoking history and comparatively good lung function. A comparison of the clinical characteristics between the COPD and non-COPD groups revealed that more patients in the COPD group were advanced in age, had a smoking history, and were diagnosed with squamous cell type, which is consistent with results of previous studies [23, 24]. A large number of comorbidities also occurred in the COPD group. Therefore, it can be inferred that patients with NSCLC and COPD had more respiratory symptoms and worse prognosis. Respiratory symptoms such as cough, sputum, and dyspnea were more frequent in patients with NSCLC and COPD than in those without, which supports the hypothesis and is consistent with the results of previous studies [25]. Declines in lung function and the severity of symptoms in patients with lung cancer are known to have significant effects on quality of life [26], and in particular, patients who suffer from dyspnea are known to be at higher risk of death and poor prognosis than those without dyspnea [6, 8]. Therefore, in this study, the quality of life in patients with advanced NSCLC was analyzed using the EORTC QLQ-C30 scale. No significant difference was found in the overall quality of life between the COPD and non-COPD groups. However, the COPD group had significantly reduced quality of life in certain aspects, such as the functional scale, cognitive and social functions, and some symptom scales, and the subgroup analysis results confirmed that overall quality of life decreased in the COPD group as the severity of airway obstruction increased. In particular, the difference was remarkable on the symptom scale related to dyspnea as well as the degree of decrease in social functioning. These results indicated that the severity of symptoms, including dyspnea, can affect various aspects of quality of life among patients with advanced NSCLC. Therefore, it is useful not only to collect baseline data regarding patient cancer status, but also to accurately evaluate patient symptoms, functional factors, presence of COPD, and severity of airway obstruction to present treatment directions for patient with lung cancer. Counselling for smoking cessation, prescription inhalers, pulmonary rehabilitation and other tailored management can improve functional status and relieve symptom burden of individual patients. Special attention by medical personnel is required.

This study also analyzed how COPD affected survival rates among patients with advanced NSCLC. Survival time was significantly shorter in the COPD group than that in the non-COPD group, which was a significant result in the univariate analysis, but not in the multivariate analysis corrected for the effects of other variables. This finding is similar to that of a previous study, which reported that the presence of COPD has no significant effect on prognosis for lung cancer patients [27]. This result suggests that it is difficult to determine the prognosis for patients with advanced NSCLC and COPD based on this single variable, because patients with COPD are more likely to have advanced stage, poor performance, and various complications during the treatment process.

This study had the following limitations. First, it was difficult to generalize the results because of the small sample size. However, the subjects were limited to patients with stage-3 and stage-4 NSCLC; those with stage-1, or stage-2 NSCLC were excluded, which had the effect of excluding other factors, such as curative surgery, that affects survival of patients. Therefore the subjects became homogeneous, which was an advantage, and the findings are highly relevant because they reveal how COPD affects symptoms, quality of life, and prognosis, even in patients with advanced lung cancer, unlike previous studies of long-term survivors after surgery [6]. Second, because the study had a retrospective design, its statistical power is rather weak. However, the questionnaire survey regarding symptoms and quality of life was done at the time of diagnosis, and objective lung functions were faithfully reflected, so the findings of this study are considered to be highly valuable for research. Third, the symptom questionnaire, pulmonary function from enrolled patients were recorded prospectively at the time of lung cancer diagnosis. Therefore, it was difficult to clearly identify the order of two diseases. However, COPD and lung cancer have a common etiology of smoking. And there are many opinions that COPD could be a driving factor in lung cancer by chronic systemic inflammation and DNA damage in time sequence [28]. We can suggest that patients with lung cancer and COPD at baseline develop more symptoms and lower quality of life than without COPD.

## Conclusions

In this study, patients with advanced NSCLC had a high prevalence rate of COPD, and patients with COPD had more symptoms, such as cough, sputum, and dyspnea, than those without COPD. Quality of life was confirmed to decrease in several aspects in patients with COPD, and overall quality of life decreased as the severity of airway obstruction increased in this group. However, no significant differences in prognosis were observed according to the presence of COPD in patients with advanced NSCLC. Based on these results, an active assessment needs to be conducted to evaluate the quality of life and symptoms in patients with lung cancer and investigate the severity of airway obstruction through pulmonary function testing at the time of the initial diagnosis. Furthermore, multilateral efforts are needed to improve the quality of life for patients with lung cancer.

## Abbreviations

AP: Appetite loss
BMI: Body mass index
CF: Cognitive functioning
CI: Confidence interval
CO: Constipation
COPD: Chronic obstructive pulmonary disease
DI: Diarrhea
DY: Dyspnea
EF: Emotional functioning
EORTC-QLQ: European organization for research and treatment of cancer quality of life questionnaire
FA: Fatigue
FEV_1_: Forced expiratory volume in 1 s
FI: Financial difficulties
HR: Hazard ratio
NSCLC: Non-small cell lung cancer
NV: Nausea and vomiting
OS: Overall survival
PA: Pain
PF: Physical functioning
QL: Global health status
RF: Role functioning
SF: Social functioning
SL: Insomnia
